# Bacterial CRISPR Regions: General Features and their Potential for Epidemiological Molecular Typing Studies

**DOI:** 10.2174/1874285801812010059

**Published:** 2018-04-23

**Authors:** Zahra Karimi, Ali Ahmadi, Ali Najafi, Reza Ranjbar

**Affiliations:** Molecular Biology Research Center, Systems Biology and Poisonings Institute, Baqiyatallah University of Medical Sciences, Tehran, Iran

**Keywords:** CRISPR, Bioengineering, Genotyping, Epidemiological studies, Pathogenic bacteria, Prokaryotic immune system

## Abstract

**Introduction:**

CRISPR (Clustered Regularly Interspaced Short Palindromic Repeats) loci as novel and applicable regions in prokaryotic genomes have gained great attraction in the post genomics era.

**Methods:**

These unique regions are diverse in number and sequence composition in different pathogenic bacteria and thereby can be a suitable candidate for molecular epidemiology and genotyping studies. Results:Furthermore, the arrayed structure of CRISPR loci (several unique repeats spaced with the variable sequence) and associated *cas* genes act as an active prokaryotic immune system against viral replication and conjugative elements. This property can be used as a tool for RNA editing in bioengineering studies.

**Conclusion:**

The aim of this review was to survey some details about the history, nature, and potential applications of CRISPR arrays in both genetic engineering and bacterial genotyping studies.

## 1 INTRODUCTION

Classification and molecular typing of pathogenic bacteria are an important issue in modern microbiology. The main purpose of microbial typing is to assess the relationships between microbial isolates These techniques have a the main role to determine the source and routes of infections, confirm or rule out outbreaks, to assess the cross-transmission of healthcare-associated pathogens, to recognize particularly virulent strains and finally to evaluate the effectiveness of control measures [1].

Actually, there is a wide variety of typing methods applied in studies of pathogenic bacteria to screening and microbial source tracking purposes. These techniques are classified into two groups, including traditional and molecular typing methods [2]. Traditional methods that consist of serotyping, phagetyping, and antibiogram typing methods have some weaknesses such as variable results, are highly time-consuming as well as have low sensitivity and specificity [3]. So, traditional methods have limitations that do not permit to understand about bacterial population genetics, evolution, and molecular epidemiology for more appropriate and accurate typing. Since 2000, high-throughput and high-resolution molecular typing methods are integrated profoundly with epidemiologic molecular studies, most of which are DNA-based and PCR-based methods [2, 3].

PCR and similar nucleotide-based methods have become potentially powerful approaches in microbial detection as well as microbial typing because of their higher user-friendliness, rapidity, reproducibility, accuracy and affordability [4, 5]. These techniques consist of analysis of plasmid profiles [6, 7], Ribotyping [8, 9], PFGE (Pulsed-Field Gel electrophoresis) [10, 11], RFLP (Restriction Fragment Length Polymorphism) [12, 13], MLST (Multi Locus Sequence Typing) [14], VNTR (Variable Number Tandem Repeat) [15, 16], RAPD (Randomly Amplification of Polymorphic DNA) [17, 18], AP-PCR (Arbitrary Pprimed PCR) [19], Rep-PCR (Repetitive extragenic palindromic) [20], ERIC-PCR (Enterobacterial Repetitive Intergenic Consensus) [21-23], Microarray [24, 25] and most recently, CRISPR regions analysis being used as a new and powerful method for molecular typing and genetic comparative analyses of bacterial strains. In this review, we aim to explain the nature, history, and epidemiological applications of CRISPR region analysis.

## 2 HISTORY OF CRISPR RESEARCH

In 1987, Ishino and colleagues recognized for the first time a new mysterious distinct class of interspaced Short Sequences Repeats (SSRs) downstream of the *iap* gene on the chromosome of *Escherichia coli K12*. These sequences comprised of repetitive motifs of 29 nucleotide identical direct repeats separated by variable 32 nucleotide spacer regions; But the biological role of these repetitive motifs remains unclear [26, 27].

In 1992, Groenen and colleagues found 36 bp repeat units being separated by 35-41-nt spacers in the genome of *Mycobacterium tuberculosis* and named it “Direct Variable Repeats” [28]. Subsequently, Mojica and coworkers identified the same repeats in *Haloferax volcanii* and *Haloferax mediterranei* and referred them as Tandem Repeats (TREPs) [29]. In 1997, Goyal and his team showed that the diversity of these spacers in *M. tuberculosis* could be used for a new specific genotyping method called Spoligotyping [30]. In 2000, Mojica *et al*. surveyed the occurrence of TREPs in a number of eubacteria and archaea and suggested that these motifs constituted a new family of prokaryotic repeats. They coined a new acronym, SRSRs (Short Regularly Spaced Repeats), to appreciate the unique regularity of these repeats [31]. Also, other names for the repeat/spacer arrays are SPacer Interspaced and Direct Repeats (SPIDRs) and Long Clustered Tandem Repeats (LCTRs) [32]. Finally, At the same time with the discovery of the first core CAS genes, Jansen *et al*. introduced “CRISPR” as a fresh acronym [33].

Although the biological function of the CRISPR regions was unknown up to that time, several activities such as developmental regulation [34], replicon partitioning during cell division [29], and DNA repair [35] were attributed to these arrayed sequences. In 2005, a profound change occurred in our understanding of the nature and function of the CRISPR regions when several independent research teams found independently that many CRISPR spacers were most often homologous to fragments of the mobile genetic elements (phages, plasmids, and transposons). It suggests that they were from the extrachromosomal origin and probably the memory of a novel prokaryotic immune system [36-39]. Subsequently, the immune system was proposed to act by using the RNA interference (RNAi) principle [40], based on the presence of some key elements that would carry out the necessary functionalities [41]. Two years later in 2007, the first experimental based evidence was provided by Barrangou *et al* [42] for the association of the CRISPR regions with the adaptive immunity in *Streptococcus thermophilus.* He indicated that acquiring new spacers are accompanied with the ability to phage resistance in a CAS-dependent manner. Soon after, it was demonstrated in *E. coli* that the CRISPR involved immunity is mediated by small noncoding interfering CRISPR RNAs (crRNAs) that direct CRISPR-associated complex for antiviral defense (cascade) [43, 44]. Furthermore, CRISPR mediated immunity against plasmid DNA was shown in *Staphylococcus epidermidis* and also supported their observation through Bioinformatics predictions that CRISPR/Cas system is an active prokaryotic immune system against phages and plasmids [45].

In 2010, numerous consecutive studies were directed to the mechanism of CRISPR regions- conferred immunity and finally concluded that CRISPR/CAS system is an active prokaryotic system immune system against viral replication and conjugative elements [46]. So, CRISPR is now considered as the hallmark of ingenious antiviral defense mechanism in prokaryotes. In 2011, three main types of classification of the different CRISPR-CAS system were suggested by Makarova *et al* in the base of the phylogeny and the presence of particular CAS proteins.

## 3 THE CRISPR REGIONS: A NEW CONCEPT

Throughout the course of evolution, prokaryotes have succeeded to use a number of innate defensive strategies against bacteriophages. An example of such systems is adsorption inhibition by which bacteria hide or modify their receptors to escape from recognition by viral particles [27, 47]. A new form of defense system has been discovered in some bacteria characterized by the presence of specific arrayed sequences named CRISPR (Clustered Regularly Interspaced Short Palindromic Repeats), which provides a defense mechanism against viruses, plasmid, and transposon [27, 48].

There are two main classes of Short Sequences Repeats (SSRs): contiguous repeats and interspersed repeats [49]. CRISPR arrays comprise two main functional elements: highly conserved 23 to 55 nucleotides tandem short DNA repeats, and 26 to 72 nucleotides variable sequences called Spacers which separate the conserved regions [37, 42]. The CAS genes are co-localized with the spacer arrays, but this confuses the origin of spacer sequences with CAS genes. The CRISPRs are varied in bacterial genomes from one strain to another and therefore, this heterogeneity may lead to phenotypic differences. These repetitive sequences are common in the genomes of prokaryotic organisms and are variable in length, sequence, and position and often are unique for a single strain, thereby making them as a good tool for the identification of specific strains of bacteria and molecular epidemiology studies.

### 3.1 The Composition of the CRISPR Region

**Fig. (1):**
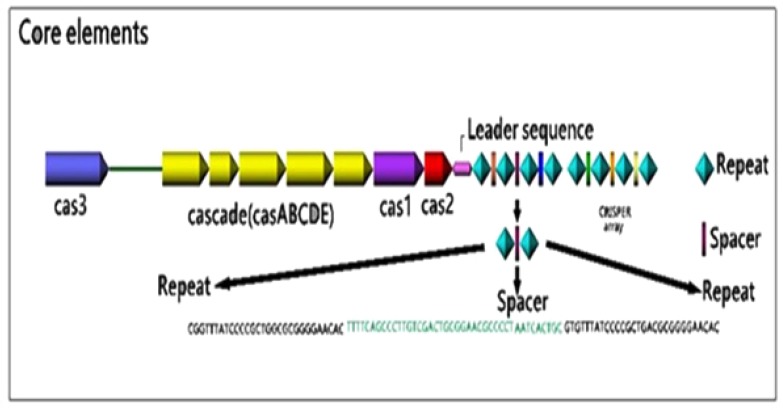
Schematic image of the CRISPR loci.

The CRISPR loci contain three elements: Direct repeated sequences; Non-repetitive spacer sequences; and a leader sequence flanking at one end of the repeats. Also, CAS genes are associated with the CRISPR loci as regulatory elements [33]. The schematic image of the CRISPR loci was illustrated in (Fig. **1**).

#### 3.1.1 Direct Repeated Sequences

The CRISPR region varies between various organisms according to the number and also the size (from 23-55 base pairs in length). These repeats that are clustered into one or more loci on the chromosome are often partially palindromic with the ability to form hairpin structures. On average, the bacterial genome contains three CRISPR arrays, compared to five CRISPR arrays found in the archaeal genome [48]. A considerable characteristic of the CRISPR arrays is the ability of their transcripts to form RNA secondary structures [50].

#### 3.1.2 Non-Repetitive Spacer Sequences

The spacer sequences vary in size from 26-72 base pairs. The spacers have similar lengths within a particular CRISPR array, but two identical spacers are not found in the same array [51]. Although it is believed that the spacer sequences have originated from foreign mobile genetic elements, just a small portion of all known spacer maps have been found to contain extrachromosomal sequences from phages and plasmids [52]. Recent CRISPR analysis studies have shown a significant polymorphism in the number and type of spacer sequences within different strains of a particular species, thereby turning them into a suitable tool for epidemiological studies. Besides, there is an offer that CRISPRs can impact the autoimmunity by use of spacers that target self-genes so CRISPRs can incur an autoimmune fitness cost by incorporation of nucleic acids, that may justify the abundance of degraded CRISPR systems across prokaryote [53].

#### 3.1.3 Leader Sequences

The AT rich, non-coding stretch nucleotides are located on one side of the CRISPR loci at the 5^ʹ^ end, upstream of the first CRISPR repeat and often downstream of the last *cas* gene [33, 54]. Leader sequences are highly similar within the same prokaryotic species but are so different in distantly related species [33]. Since regions being homologous to the 3^'^-end of the leader sequence were found in the precursor CRISPR RNA (pre-crRNA) of *Pyrococcus furiosus,* it is suggested that the transcription of the CRISPR arrays is initiated in the leader region [55]. Recently, the role of leader sequences as a promoter for transcription of the pre-crRNA has been demonstrated. Besides, it has also been suggested that the leader sequences could prepare a platform for binding of the CAS proteins required for integrating the spacers [36, 46]. This promoter was active both *in vitro* and *in vivo* and was able to form an open transcription initiation complex [56]. Bioinformatics analysis has shown that CRISPR loci lacking leader sequences are unable to incorporate new spacers [46] and to execute the CRISPR-Expression and Interference [45]. The direct repeats and leader sequences are both conserved in a single bacterial species but are diverse between different species.

#### 3.1.4 CAS Genes

CRISPR-Associated (CAS) genes have been only detected in CRISPR-containing prokaryotes and have invariably been located adjacent to the CRISPR loci [57]. It is suggested that CRISPR loci and CAS genes have related functions, especially in DNA metabolism and/or gene expression [44].

Haft and coworkers in 2005 documented 45 CAS gene families constituting six core CAS families (*cas*1-*cas*6), among which two families (*cas*1 and *cas*2) are universal and present in all CAS subtype [58, 59]. *Cas*1and *cas*2 families are implicated in the novel spacer acquisition, novel repeat synthesis, and repeat-spacer insertion at the leader end [44].

Furthermore, three CRISPR-CAS type systems have been recently recognized based on the phylogeny and also the molecular mechanism of action of *cas* genes, including type Ι, according to the presence of *cas*3 gene; type ΙΙ, for the presence of *cas*9 gene; and type ΙΙΙ, for the presence of *cas*10 gene [60, 61]. Although type Ι and type ΙΙΙ CRISPR-CAS system have some common features, it is not the case for the type ΙΙ system. Aside from a conserved set of *cas* genes (*i.e.*, *cas*1, *cas*2, and *cas*9), three different subtypes have been identified, including type ΙΙ-A with an additional *cas*2, type ΙΙ-B type ΙΙ-C with an additional *cas*4, and type ΙΙ-C with no additional gene [60].

Cas proteins comprise a highly genetically polymorphic and functionally diverse family that is involved in various stages of CRISPR-mediated immunity. Several different Cas protein families, being highly variable in number, distribution, and also organization has been classified [60, 62]. The most widely distributed functional domain that is characteristic of Cas proteins is the RNA Recognition Motif (RRM). Based on sequence analysis studies, nucleases, helicases, integrases, and polymerases domains have been predicted in Cas proteins, indicating their involvement in the nucleic acid metabolism [41]. The key conserved Cas proteins and their function are listed in Table (**1**).

## 4 IN SILICO ANALYSIS OF CRISPR

### 4.1 CRISPR databases

**Fig. (2):**
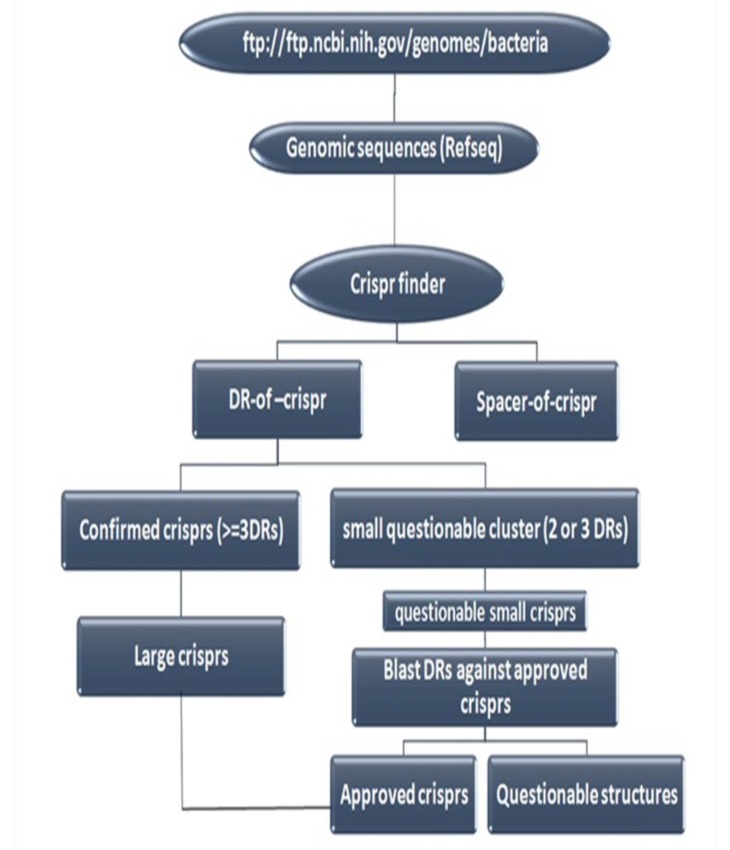
A flow chart for discovering CRISPR regions in the bacterial genome.

Bioinformatics and their capabilities can accelerate molecular epidemiologic investigations in post genomics era. In this regard, several special CRISPR databases and tools have been developed during recent years. There are two general categories of CRISPR database: 1) Gene Editing based databases, and 2) Genotyping based databases. The second group (Genotyping based databases) is a database that deposits CRISPR sequences and their annotations. CRISPR Finder [63] and CRISPRdb [48] are the best examples of Genotyping based databases that are specific resources for deposition of CRISPR loci and spacers in some prokaryotic genome. But, since Genotyping based databases do not cover all bacterial genomes, a flowchart designed here as (Fig. **2**) could be used for discovering CRISPR regions in bacterial and archeal complete genomes [48].

### 4.2 CRISPR Tools

Despite the presence of several various bioinformatic tools for identifying direct repeats in the genome, due to the importance and nature of CRISPR composition, specific software applications have been developed for discovering them in prokaryotic genomes and metagenomes including CRISPR Finder, CRISPR Recognition tool (CRT), PILER-CR, and CRISPI.

Among them, the CRISPR Finder is freely accessible at http://crispr.u-psud.fr/crispr/, a user-friendly web service that contains information about CRISPR systems in several bacterial genomes which can identify and extract CRISPR array and spacers. This program can find the largest number of possible CRISPRs, especially the shortest ones that only contain one or two spacers [63].

## 5 ROLE OF CRISPR-CAS SYSTEM

**Fig. (3):**
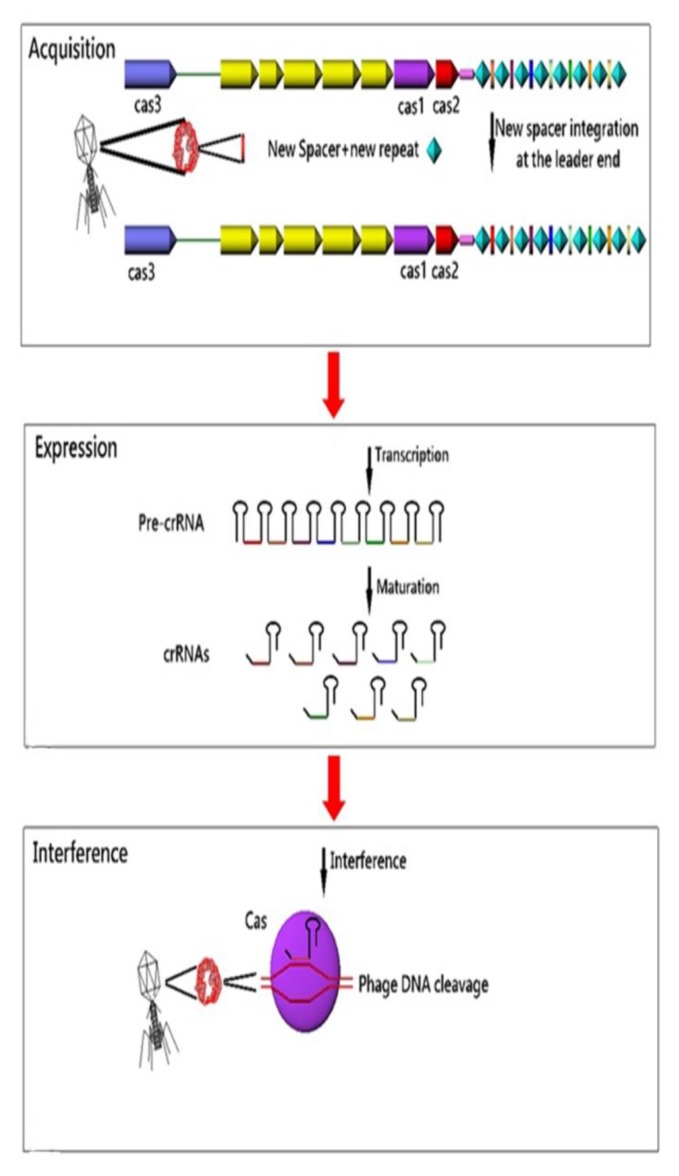
Development stages of the CRISPR/CAS system.

CRISPR arrays and *cas* genes are the two parts of the CRISPR-CAS immune system in bacteria and archaea, which provides adaptive immunity against foreign genetic elements. Conferring of immunity is the duty of spacers. These immune markers are transcribed and processed into small non-coding interfering CRISPR RNAs (crRNAs) that direct Cas proteins toward foreign nucleic acids for specific cleavage of homologous sequences [41]. In fact, it has been suggested that CRISPR-CAS system memorizes invaders by incorporating the new invader-derived DNA sequences into the CRISPR sequences and providing immunity to future attack by the same invader [40]. Overall, CRISPR-CAS system functions in three steps: 1) Adaptation: new spacers are acquired from invader nucleic acids and integrated into the CRISPR loci; 2) Expression: CRISPR loci are transcribed and processed into small interfering crRNAs; and 3) Interference: cr-RNAs direct the CAS machinery to specifically cleave homologous invader nucleic acids [57]. The response cascade of the CRISPR/CAS system is shown in (Fig. **3**). In 2009, Zegans *et al* suggested that the CRISPR regions can change the lysogeny effects of Pseudomonas aeruginosa PA14 by restoring both the biofilm formation and swarming motility, which had been inactivated by lysogenic bacteriophage DMS3 [64]. Also, Edgar and Qimron have shown that the CRISPR system protects bacteria during the lysogenic cycle of phages [65].

### 5.1 CRISPR-Cas 9 Editing

The CRISPR-Cas 9 is a RNA-guided genome editing tool for genome editing purposes such as gene therapy studies and therapeutic purposes in cell lines or animal models.

It can act for the correction of causal mutations in monogenic disorders, or manipulate pathogen genomes such as HIV, or induce protective or therapeutic mutations in host tissues. Also, the potential of CRISPR-CAS 9 for deactivating oncogenic viruses and inducing oncosuppressor expressions for cancer gene therapy has been shown [66].

## 6 OCCURRENCE AND EPIDEMIOLOGICAL APPLICATIONS OF CRISPR SYSTEM

During the past decade, studies have shown that CRISPR regions are a family of repetitive sequences that are present in the genome sequence of approximately 48% of bacteria and 90% of archaea. In contrast, they have not been identified in eukaryotic genomes and also viruses to date. Gram positive and negative bacteria found to harbor CRISPR regions are listed in Table (**2**) [33].

Some bacterial strains may have spacer sequences with different numbers at the same CRISPR loci, offering a strain-specific polymorphism that can be used in phylogeny and epidemiological studies [63]. But, it is noteworthy that since some bacterial species (about 50%) did not acquire CRISPR loci in their genomes, and also some bacterial species acquired spacers at a higher rate, they could not be sub-grouped through the CRISPR based- molecular subtyping [3]. Spacer-oligonucleotide typing or “Spoligotyping” was the first use of spacer information for bacterial subtyping applications [28, 88]. The principle is the PCR amplification of the CRISPR arrays with labeled primers that detect direct repeat sequences, followed by hybridization of the PCR products to a membrane containing probes bearing spacer DNA sequences. Because of strain-specificity, different hybridization patterns could distinguish between different strains [3]. For example, Simon Le Hello and coworkers in 2013 reported that CRISPR sequences are very applicable targets for subtyping of *Salmonella enteritidis* isolates. They concluded that since CRISPR spacer content can be easily obtained from short-read DNA sequences, it could be used to identify particular *Salmonella* and probably other bacterial pathogens [89, 90]. Also, Touchon *et al*. analyzed the CRISPR loci of 51 complete genomes of *Salmonella* and *Escherichia* isolates and found two pairs of CRISPR loci in Escherichia and one single pair in *salmonella*. In general, different studies in recent years have shown controversial results for CRISPR loci based bacterial genotyping. For example, a study on *Campylobacter jejuni* isolates concluded that CRISPR based genotyping is not an appropriate approach for Campylobacter species, because some campylobacter species either lacked an amplifiable CRISPR locus or contained just a single DR [3]. Also, in two separated studies, it has been shown that CRISPR loci had a few changes in the genome of Shigella species [85], and are not widely distributed in *Klebsiella pneumoniae* isolates too, thereby making CRISPR loci a poor genotyping marker for these two pathogens [86]. Some studies, on the other hand, have found promising results, especially for *Salmonella* isolates. A study on *Salmonella enterica* serotypes typhi and paratyphi has shown that CRISPR regions could be used as an original target for the development of PCR assays specific for particular *salmonella* species [91]; another study comparing the CRISPR based genotyping with PFGE and MLVA techniques observed high levels of correlation between their results [3]. Furthermore, some researchers have suggested that the CRISPR/CAS system may be an important tool for evolutionary dynamics investigations for Clostridium difficile isolates [71].

Conclusion: The CRISPR loci have potential for broad genotyping of bacterial strains and could be further used in an epidemiological survey [41]. CRISPR features can, moreover, be used for host-virus environmental studies, offering a specific immunity against undesirable genetic elements, and increasing viral resistance in domesticated microbes [57]. By use of CRISPR loci as a template for genotyping, we retrieve information more precisely to define the epidemic strains, because the spacers inserted in a polarized manner at the leader end of CRISPR loci can lead to provide a genetic basis for the detection of historical path of a strain, share ancestry between strains and establish phylogenetic relationships. So, these hypervariable loci of the CRISPR with novel Bioinformatics tools for processing of high-throughput sequencing data, and the visualization of complex datasets are broadly useful for epidemiological surveys.

## Figures and Tables

**Table 1:** Key conserved Cas proteins and their function.

**Cas protein**	Function
**Cas 1**	Specifically exhibits nuclease activity against single stranded and branched DNA, replication forks, and may be implicated in addition of novel repeats and/or spacers.
**Cas 2**	Involved in novel spacer acquisition, novel repeat synthesis and repeat-spacer insertion at the leader end.
**Cas3** **(signature gene for type I)**	Encodes a nuclease involved in the cleavage of the target DNA so responsible for the processing of crRNA and involved in recognition of target DNA.
**Cas 6** **(key protein for type Ι)**	An endoribonuclease which cleaves the pre-crRNA within the CRISPR repeat sequence during the crRNA maturation process.
**Cas 9** **(signature gene for type ΙΙ)**	Encodes a large protein involved in both crRNA biogenesis and target DNA cleavage.
**Cas 10** **(signature gene for type III)**	Encodes a nuclease implicated in target nucleic acid interference.

**Table 2:** Gram positive and negative bacteria harboring CRISPR regions.

**Organism**		**Ref**
Gram positive	*Enterococcus spp*	Lyons *et al*., 2015 [67]
	*Streptococcus thermophilus*	Shariat *et al*., 2015 [68] Barrangou *et al*., 2007 [42]
	*Streptococcus pneumoniae*	Bikard *et al*., 2012 [69]
	*Streptococcus pyogenes*	Zheng *et al*., 2015 [70]
	*Clostridium difficile*	Hargreaves *et al*., 2014 [71] Andersen *et al*., 2016 [72]
	*Lactobacillus buchneri*	Briner *et al*., 2014 [73]
	*Staphylococcus epidermidis*	Marraffini *et al*., 2008 [74]
	*Listeria monocytogenes*	Di, Huiling *et al*., 2014 [75]
	*Corynebacterium diphtheria*	Mokrousov *et al*., [76-78]
Gram negative	*Campylobacter spp*	de Cárdenas, Inés *et al*., 2015 [79]
	*Yersinia spp*	Pourcel C *et al*., 2005 [80]
	*Escherichia coli*	Ishino *et al*., 1987 [26] Brouns *et al*., 2008 [43] Pul *et al*., 2010 [56] Diez-Villasenor *et al*., 2010 [81]
	*Salmonella enterica*	Fabre *et al*., 2012 [82] Li *et al*., 2014 [83] Le Hello *et al*., 2015 [84] Shariat *et al*., 2015 [68]
	*Shigella*	Guo *et al*., 2015 [85]
	*Klebsiella pneumoniae*	Ostria *et al*., 2015 [86]
Acid Fast Bacilli	*Mycobacterium spp*	Groenen *et al*., 1993 [28] Goyal *et al*., 1997 [30] Sola *et al*., 2015 [87]
